# Comparative Evaluation of Bovine- and Porcine-Deproteinized Grafts for Guided Bone Regeneration: An In Vivo Study

**DOI:** 10.3390/bioengineering12050459

**Published:** 2025-04-26

**Authors:** Blaire V. Slavin, Vasudev Vivekanand Nayak, Marcelo Parra, Robert D. Spielman, Matteo S. Torquati, Nicholas J. Iglesias, Paulo G. Coelho, Lukasz Witek

**Affiliations:** 1 University of Miami Miller School of Medicine, Miami, FL 33136, USA; 2 Department of Biochemistry and Molecular Biology, University of Miami Miller School of Medicine, Miami, FL 33136, USA; 3 Dr. John T. Macdonald Foundation Biomedical Nanotechnology Institute (BioNIUM), University of Miami, Miami, FL 33136, USA; 4 Department of Comprehensive Adult Dentistry, Faculty of Dentistry, Universidad de La Frontera, Avenida Francisco Salazar 01145, Temuco 4811230, Chile; 5 Center of Excellence in Morphological and Surgical Studies (CEMyQ), Faculty of Medicine, Universidad de La Frontera, Avenida Francisco Salazar 01145, Temuco 4811230, Chile; 6 Biomaterials and Regenerative Biology Division, NYU College of Dentistry, New York, NY 10010, USA; 7 CTOR Academy, Hoboken, NJ 07030, USA; 8 DeWitt Daughtry Family Department of Surgery, University of Miami Miller School of Medicine, Miami, FL 33136, USA; 9 Division of Plastic Surgery, DeWitt Daughtry Family Department of Surgery, University of Miami Miller School of Medicine, Miami, FL 33136, USA; 10 Department of Biomedical Engineering, NYU Tandon School of Engineering, Brooklyn, NY 11201, USA; 11 Hansjörg Wyss Department of Plastic Surgery, NYU Grossman School of Medicine, New York, NY 10016, USA; 12 Department of Oral and Maxillofacial Surgery, NYU College of Dentistry, New York, NY 10010, USA

**Keywords:** guided bone regeneration, xenografts, anorganic bone, alveolar ridge preservation

## Abstract

Guided bone regeneration (GBR) procedures have been indicated to enhance bone response, reliably regenerate lost tissue, and create an anatomically pleasing ridge contour for biomechanically favorable and prosthetically driven implant placement. The aim of the current study was to evaluate and compare the bone regenerative performance of deproteinized bovine bone (DBB) and deproteinized porcine bone (DPB) grafts in a beagle mandibular model for the purposes of GBR. Four bilateral defects of 10 mm × 10 mm were induced through the mandibular thickness in each of the 10 adult beagle dogs being studied. Two of the defects were filled with DPB, while the other two were filled with DBB, after which they were covered with collagen-based membranes to allow compartmentalized healing. Animals were euthanized after 6, 12, 24, or 48 weeks postoperatively. Bone regenerative capacity was evaluated by qualitative histological and quantitative microtomographic analyses. Microcomputed tomography data of the bone (%), graft (%), and space (%) were compared using a mixed model analysis. Qualitatively, no histomorphological differences in healing were observed between the DBB and DPB grafts at any time point. By 48 weeks, the xenografts (DBB and DPB) were observed to have osseointegrated with regenerating spongy bone and a close resemblance to native bone morphology. Quantitatively, a higher amount of bone (%) and a corresponding reduction in empty space (space (%)) were observed in defects treated by DBB and DPB grafts over time. However, no statistically significant differences in bone (%)were observed between DBB (71.04 ± 8.41 at 48 weeks) and DPB grafts (68.38 ± 10.30 at 48 weeks) (*p* > 0.05). GBR with DBB and DPB showed no signs of adverse immune response and led to similar trends in bone regeneration over 48 weeks of permitted healing.

## 1. Introduction

The catabolic changes observed after tooth loss are initiated by the resorption of the bundle bone, a tooth-dependent structure that lines the extraction socket [1]. Due to its dependence on tooth alterations, the bundle bone is gradually resorbed following tooth extraction, which leads to bone loss on the facial aspect, which contrasts the minimal bone resorption observed on the lingual aspect [1]. Various conditions can lead to tooth loss, ultimately contributing to bone atrophy in the affected region. These conditions encompass, but are not restricted to, extensive carious lesions, periapical pathology, dental fractures, and/or periodontal disease [2,3,4,5]. Furthermore, within 6 months following tooth loss or extraction, the alveolar ridge suffers a mean horizontal reduction of ~3.8 mm and a mean vertical reduction of ~1.24 mm [6]. The maintenance of bone dimensions of the alveolar ridge therefore remains a clinical challenge. Rehabilitating these edentulous areas through endosteal implants often necessitates treatment involving ridge augmentation, either before or during implant placement. Guided bone regeneration (GBR) stands out as a widely employed treatment modality for this purpose [7,8].

GBR commonly incorporates the application of particulate bone grafts or substitutes within a confined space to augment bone formation and prevent collapse of the tented site [9]. Simultaneously, a membrane is employed to secure the exclusion of soft tissue from the defect site, enabling compartmentalized hard tissue healing within the defect [10]. This helps accomplish successful bone formation and inhibits fibrogenesis originating from the surrounding soft tissue [11]. The relationship between a graft and host tissue can be subdivided into osteoinduction, the recruitment and stimulation of cells to create new bone; osteoconduction, osteogenesis on a graft surface; and osseointegration, stabilization of a graft via direct bone-to-graft contact [12]. Numerous factors, including graft composition, can significantly impact the interaction between the graft and the host’s tissue, along with the ensuing cellular response [13]. Consequently, there is a growing emphasis among the researchers and clinicians on comprehensively understanding the origins of these variations. In the present clinical scenario, autologous bone grafts persist as the recognized ‘gold standard’ [14,15]. However, due to certain limitations, such as donor site morbidity and limited quantities available for harvesting, allografts and xenografts serve as viable alternatives [14]. While allografts have been utilized in regenerative therapies, concerns surrounding osteoinductivity, risks of immunological rejection, and disease transmission have been described in the literature [16]. On the other hand, while xenografts also require similar processing steps to allografts for the removal of unwanted organics, they have distinct advantages [17].

Xenografts, particularly deproteinized bone grafts, exhibit a decreased risk of infections and cost-effectiveness compared to alloplasts [17,18]. Furthermore, they present a higher level of availability relative to autografts [18]. Deproteinized bone grafts also share many similarities with human bone, such as similar calcium/phosphorous (CaP) content and morphology [18]. Among the diverse deproteinized grafts employed for GBR, those derived from bovine sources have become prevalent in clinical practice [19,20,21,22]. Deproteinized bovine bone (DBB) grafts are distinguished by their osteoconductive properties and a slow resorption rate, thereby aiding in the preservation of bone volume [23]. In fact, DBB particles have been observed to remain largely intact several years post-implantation, underscoring their long-term volume stability [24]. Among the various bovine-derived grafts available for clinical application, Bio-Oss (Geistlich-Pharma, Wolhunsen, Switzerland) stands out as a xenogeneic material that closely mimics the chemical composition of native bone [17]. The processing steps of Bio-Oss have been described to involve sintering at 300 °C and treatment with a strongly alkaline solution, which deproteinizes the bovine bone and removes organic components [25]. Bio-Oss has been extensively used in both basic and clinical dentistry as a xenograft material for alveolar ridge preservation and sinus lift procedures [22,26,27].

On the other hand, porcine-derived bone (DPB) grafts have been more recently developed. They are considered to exhibit similarities in structure and composition to human bone due to the shared characteristics in the human and porcine genomes [28]. Furthermore, they exhibit osteoconductive characteristics and present a low risk of disease transmission [29,30]. While porcine-derived products are available in the market for oral surgery applications, there is a scarcity of data reporting their osteoconductive effectiveness. Thus, the aim of the current study was to evaluate and compare the bone regenerative performance of DBB and DPB grafts in a large translational (beagle) mandibular model, providing long-term in vivo data to inform graft selection for potential clinical application.

## 2. Materials and Methods

### 2.1. Bone Grafts

A DBB graft (Bio-Oss Collagen) was procured from Geistlich Pharma AG (Wolhusen, Switzerland) and comprised of 90% DBB (small cancellous bone granulates: 0.25–1 mm) in a 10% collagen matrix. On the other hand, an experimental porcine-derived particulate graft (DPB) was provided by Regenity Biosciences (Oakland, NJ, USA) and comprised of 100% DPB granules (0.25–1 mm) [31]. In brief, the DPB graft comprised of porous, anorganic bone mineral with a carbonate apatite mineral structure, derived from porcine cancellous bone, and presented a white-to-off-white appearance. Both DBB and DPB grafts were synthesized by proprietary treatment protocols, including multi-stage purification processes by the manufacturers, and were used directly as supplied.

### 2.2. Surgical Procedure

Following approval from the Institutional Animal Care and Use Committee (protocol#: IAN001-IS75), surgery was performed on *n* = 10 adult beagle dogs (~1.5 years of age) at American Preclinical Services (APS, Minneapolis, MN, USA). The animals were housed in individual kennels and allowed to acclimate for 14 days before surgery. Animals were fasted overnight before surgery to prevent vomiting during the administration of general anesthesia. As per the approved protocol, animals were anesthetized by the intramuscular (IM) administration of 0.1–0.2 mg/kg midazolam 1×, 0.05–0.1 mg/kg butorphanol 1×, and an intravenous (IV) administration of 2–8 mg/kg propofol to effect. Animals were monitored until unconscious and then prepared for surgery. During surgery, anesthesia was maintained with isoflurane (0–5%) administered via an endotracheal tube. Supplemental anesthesia (2–8 mg/kg propofol, to effect) and antiarrhythmics (0.5–2.0 mg/kg lidocaine, over 1–2 min, to effect) were administered via IV as needed.

**Figure 1:**
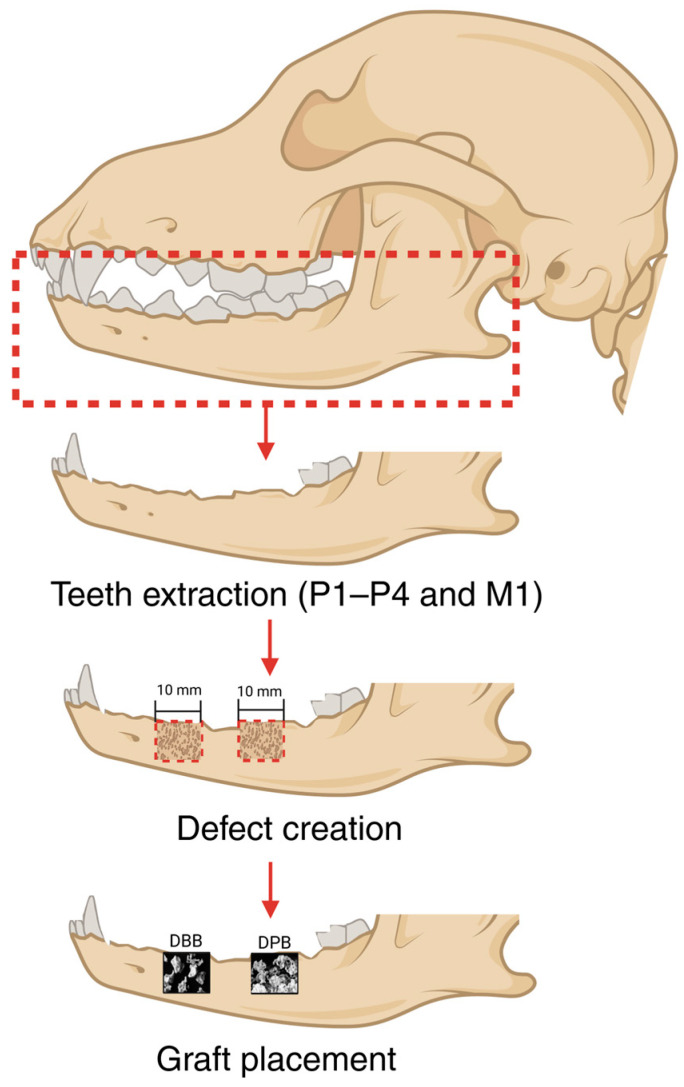
Representative schematic of the surgical aspect depicting teeth extraction, defect creation, and graft (image courtesy Regenity Biosciences and Geistlich Pharma AG) placement within the induced defects. Image not to scale. Created on BioRender.

The surgical procedure (Figure 1) commenced with the bilateral extraction of lower premolars (P1–P4) and first molar (M1). A mid-crestal incision was then made bilaterally, and a full-thickness flap was raised to expose the mandibular bone. Subsequently, two bilateral defects of 10 mm × 10 mm were created through the mandibular thickness, allowing for a lingual wall of thickness comparable to the alveolar socket’s lingual plate. This was achieved using a low-speed cylindrical burr under copious irrigation. The same procedure was replicated on the contralateral side of the mandible. Two of the defects were filled with DBB, while the other two were filled with DPB. Defects were then covered with a collagen-based barrier dental membrane to ensure compartmentalized healing and sutured closed using polyglycolic acid and polycaprolactone copolymer (PGA/PCL) sutures. All the experimental groups were nested within the animals and interpolated among defect sites to minimize site bias.

Post-operative opioid analgesia was provided by IM injections of buprenorphine sustained release (0.03–0.06 mg/kg) for 3 days, and tramadol (4–10 mg/kg) as required. To prevent postoperative infections, antimicrobial/infection prophylaxis (cefpodoxime, 5–10 mg/kg) was administered for 10 days. In consultation with the approved veterinarian, soft food was offered to the animals until evidence of incision healing. Post-incision healing, animals were fed with fixed-formula diets. Water was offered to the animals in the postoperative period ad libitum and all the animals were under constant observation for signs of postoperative complications or infections. Animals were euthanized after 6, 12, 24, or 48 weeks post-surgically by means of anesthesia overdose using isoflurane (0–5%, in 100% O_2_ via inhalation, to effect), propofol (2–8 mg/kg, via IV, to effect), heparin (20,000 IU, 1×, via IV), and euthanasia solution/barbiturate (via IV, to effect). Mandibles, including the grafted regions, were harvested by sharp dissection.

### 2.3. Volumetric Reconstruction and Analysis

Specimens were fixed in 10% formalin after euthanasia and were subsequently trimmed down using a bandsaw and stored in 70% ethanol. They were then scanned using a micro-computed tomography (microCT) apparatus (μCT 40, Scanco Medical, Basserdorf, Germany) at 70 kV and 114 μA, at a voxel resolution of 18 μm. The Digital Imaging and Communications in Medicine (DICOM) files from the scans were imported into a 3D reconstruction and visualization software, Amira 6.3.2 (Thermo Fisher Scientific, Waltham, MA, USA). Images were segmented with a predetermined threshold, which also involved isolating and extracting the region of interest from the complete dataset based on the Hounsfield Units (HUs). Quantitative analysis was performed on the segmented data to record the percent volume of bone (bone (%)), graft (graft (%)), and empty space (space (%)) within the defect sites at various time points, as per a previously established protocol [32]. All volumetric reconstruction analyses were performed by a single, blinded, and trained investigator to eliminate bias.

### 2.4. Histologic Preparation and Analysis

Samples were dehydrated progressively in ethanol (70% and 90% *v*/*v* for 24 h at each step and 100% *v*/*v* for 48 h) followed by a clearing agent, namely, methyl salicylate for 48 h prior to final embedding in methylmethacrylate. The samples were sectioned across the buccolingual plane of the mandibles using a low-speed precision diamond saw (Isomet 2000, Buehler Ltd., Lake Bluff, IL, USA) into sections of ~300 μm thickness. Each tissue section was glued to a glass slide with a low-viscosity, fast-curing, cyanoacrylate adhesive (Henkel Loctite 408 adhesive, Dusseldorf, Germany). Slides were left to dry overnight before grinding under abundant water irrigation on a polishing wheel (Metaserv 3000, Buehler Ltd., Lake Bluff, IL, USA). Silicon carbide (SiC) abrasive (400, 600, 800, and 1200 grit) papers were progressively utilized to achieve a final thickness of ~100 μm. Slides were then polished using a microfiber cloth under a copious supply of micro-polish alumina particle suspension (1.0 μm MicroPolish^TM^, Buehler Ltd., Lake Bluff, IL, USA). The samples were subsequently stained with Stevenel’s Blue and Van Gieson’s Picro Fuschin (SVG). Cells and extracellular structures stained with Stevenel’s blue exhibited blue and blue–green hues. Van Gieson’s picro-fuchsin counterstain imparted green–blue tones to collagen fibers; red to bone; yellow–green to osteoid material; and blue/blue–green to muscle fibers. This staining combination enabled the differentiation between the graft particles, hard and soft tissue structures. Histological images were obtained using an automated slide scanning system (Aperio Technologies, Vista, CA, USA) and qualitatively analyzed by a trained and experienced investigator.

### 2.5. Statistical Analysis

An a priori power analysis was conducted to determine the sample size. A minimum of 24 samples or defects was required to achieve statistical power greater than 0.8, with an effect size of 0.25, and type I error rate of 0.05. In addition, the large size of the beagle mandible facilitated multiple defects to be nested within each animal (4 defects per animal), thereby increasing the statistical power. MicroCT data of bone (%), graft (%), and space (%) were compared using a mixed model analysis (IBM SPSS v29, IBM Corp., Armonk, NY, USA) with fixed factor variables of time and material. The data from statistical analyses were presented as mean values with corresponding 95% confidence intervals (mean ± 95% CI), with *p* ≤ 0.05 being statistically significant.

## 3. Results

### 3.1. Qualitative Histological Evaluation

**Figure 2:**
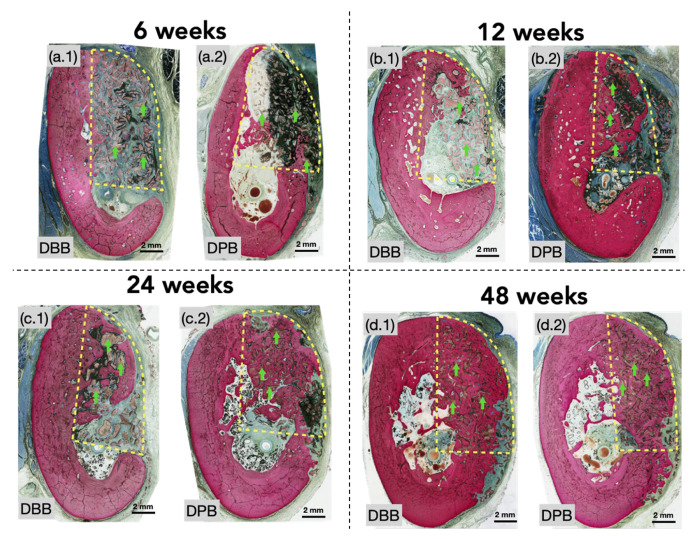
Representative histomicrographs of the defect sites treated with (**a.1**,**a.2**) DBB and DPB grafts, respectively, at 6 weeks; with (**b.1**,**b.2**) DBB and DPB grafts, respectively, at 12 weeks; with (**c.1**,**c.2**) DBB and DPB grafts, respectively, at 24 weeks; with (**d.1**,**d.2**) DBB and DPB grafts, respectively, at 48 weeks. Dashed yellow splines represent the region of interest (defect site), and green arrows point to a few of the graft particles with the defect.

**Figure 3:**
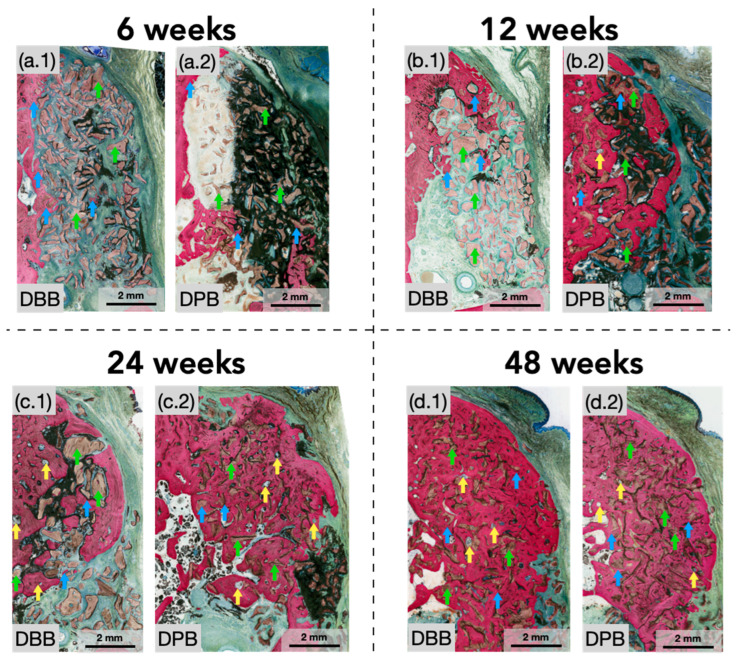
Representative high-magnification histomicrographs of the defect sites treated with (**a.1**,**a.2**) DBB and DPB grafts, respectively, at 6 weeks; with (**b.1**,**b.2**) DBB and DPB grafts, respectively, at 12 weeks; with (**c.1**,**c.2**) DBB and DPB grafts, respectively, at 24 weeks; with (**d.1**,**d.2**) DBB and DPB grafts, respectively, at 48 weeks. Green arrows represent a few of the graft particles with the defect, blue arrows represent bone formation engrossing graft particles (on their respective surfaces), and yellow arrows depict bone remodeling sites.

No signs of complications, infections, or clinical concerns were observed during follow-ups. Qualitative histological analysis of defects at 6 weeks illustrated newly formed bone extending from the periphery of defects. Graft particles at this early healing time point were predominantly surrounded by fibrovascular connective tissue and inflammatory cells. However, graft particles near the defect’s edges and a few in the defect center were also beginning to be engrossed by newly forming bone (Figure 2(a.1,a.2) and Figure 3(a.1,a.2)). Between 6 and 12 weeks, a greater degree of bone formation was appreciable in both groups with progressive osseointegration of the xenograft (Figure 2(b.1,b.2) and Figure 3(b.1,b.2)) (blue arrows), newly formed bone, and indications of early signs of bone remodeling (yellow arrows). At this healing time point (12 weeks), the extracellular matrix surrounding graft particles was more frequently replaced by regenerated bone. Although visible in both groups at 12 weeks, this phenomenon was better depicted in the DPB group. A similar progression was detected between 12 and 24 weeks, in which regenerated bone could be seen further encompassing the granule surfaces within the periphery and center (Figure 2(c.1,c.2) and Figure 3(c.1,c.2)), with little remaining soft connective tissue. By this study’s endpoint at 48 weeks (Figure 2(d.1,d.2) and Figure 3(d.1,d.2)), the xenografts (DBB and DPB) were observed to have been completely surrounded by regenerating spongy bone with a resemblance to the native bone morphology, consistent with advanced stages bone remodeling and appositional bone growth. No other histomorphological differences in healing were observed during the course of this study between the DBB and DPB grafts at any of the time points evaluated.

### 3.2. MicroCT and Volumetric Reconstruction Analysis

**Figure 4:**
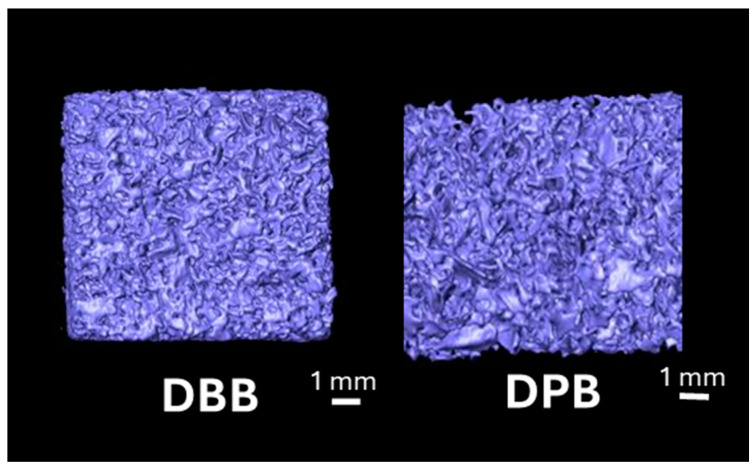
Representative volumetric reconstruction of the DBB and DPB grafts at T = 0 weeks (pre-implantation). The graft particles are highlighted in purple.

**Figure 5:**
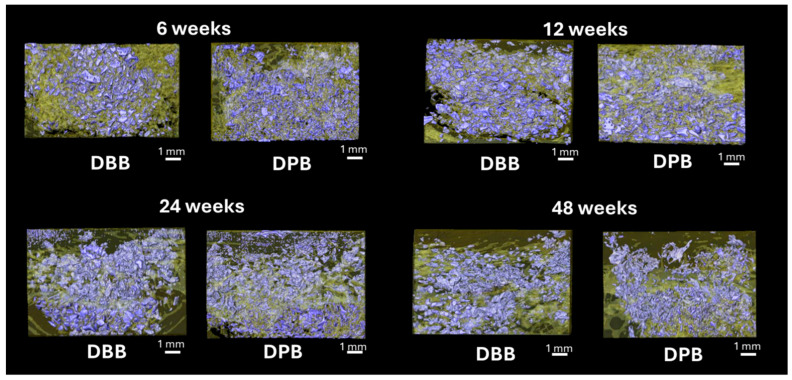
Representative volumetric reconstruction of the defect sites treated with DBB and DPB grafts after 6, 12, 24, and 48 weeks of permitted healing, with graft particles in purple and newly forming and regenerating bone in yellow.

**Figure 6:**
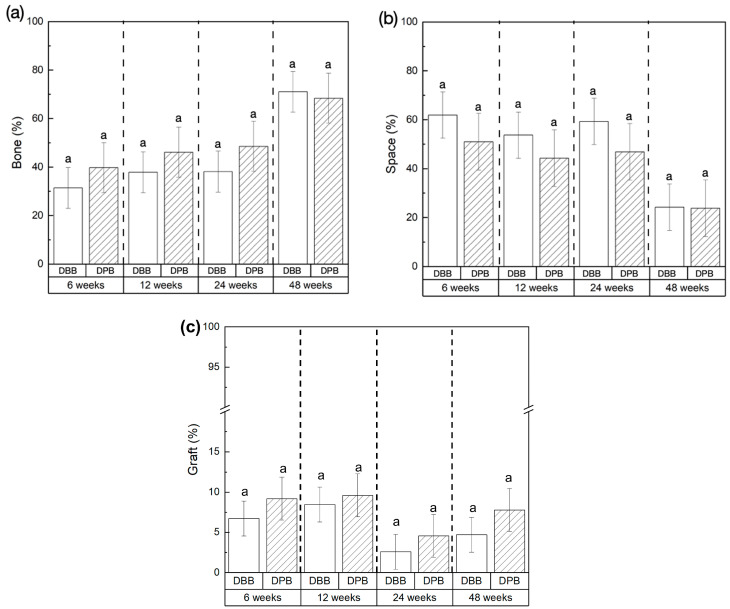
Quantification of (**a**) bone (%), (**b**) space (%), and (**c**) graft (%), within the grafted mandibular defects at 6, 12, 24, and 48weeks in vivo, presented as mean  ±  95% confidence intervals (mean  ±  95% CIs). The letters atop the error bars represent statistically homogenous groups.

Figure 4 shows the volumetric reconstruction of the particulate grafts prior to implantation (T = 0 weeks). Figure 5 highlights the volumetric reconstruction of the defect sites treated with DPB and DBB grafts after 6, 12, 24, and 48 weeks of permitted healing. A summary of the data is presented in Table 1. Over time (between 6 and 48 weeks), a higher amount of bone (bone (%), Figure 6a) and corresponding reduction in empty space (space (%), Figure 6b) was observed in defects treated by both DBB and DPB grafts. Nonetheless, no statistically significant differences in bone (%) and space (%) were observed between DBB and DPB grafts at any of the time points evaluated (*p* > 0.05). Similarly, the statistical evaluation of the remaining graft material (graft (%), Figure 6c) within the defect showed no significant differences between the groups at any time point (*p* > 0.05).

## 4. Discussion

GBR procedures have been studied to improve bone response and predictably regenerate lost tissue, providing an anatomically pleasing ridge contour for biomechanically favorable and prosthetically driven implant placement. The selection of an appropriate grafting material plays a crucial role in the long-term success of GBR, influencing bone regeneration dynamics, osseointegration, and overall clinical outcomes. Xenografts, such as DBB and DPB, have gained prominence due to their osteoconductive properties and ability to serve as a scaffold for new bone formation while minimizing the drawbacks associated with autografts and allografts [11,33,34,35,36]. With the evidence presented in this study and in the literature, it is important for clinicians to be informed about the long-term outcomes stemming from graft material selection. The current investigation presents compelling evidence of the efficacy of GBR by utilizing two distinct classes of xenografts within a preclinical canine (beagle) mandibular model. Canine models have been considered ideal preclinical models for regenerative device testing and evaluation in various studies pertaining to periodontal bone regeneration [37,38,39,40]. Their dentoalveolar similarity to the human mandible and bone turnover rate makes them a valuable translational model for assessing GBR outcomes [37,38,39,40].

A limited number of preclinical and clinical studies have directly compared DPB and DBB, generally reporting that porcine-derived xenografts achieve outcomes comparable to those of bovine-derived grafts [24,41,42]. For example, in a rabbit calvaria defect model, no statistically significant differences in new bone formation were observed between DPB- and DBB-grafted sites after 8 weeks of healing [42]. Similarly, in a rabbit maxillary sinus augmentation model, both DPB and DBB supported new bone regeneration [41]. Notably, the porcine graft underwent faster resorption, resulting in greater graft volume reduction and a higher new bone content adjacent to native bone walls [41]. Furthermore, a rat calvaria defect study evaluating hydroxyapatite mixed with demineralized bone matrix from porcine and bovine sources found that porcine-derived grafts achieved superior tissue regeneration, with significantly higher tissue thickness and bone formation after 3 months [24].

In addition to these preclinical findings, a randomized clinical trial on humans found no significant differences in bone formation or residual graft material when comparing porcine versus bovine xenografts for alveolar ridge preservation, with both groups showing comparable dimensional stability of the ridge after healing [43]. Nevertheless, the comparative evidence remains limited, particularly with regard to the long-term in vivo performance, underscoring the need for further validation. Consistent with the literature, both DBB and DPB grafts demonstrated positive outcomes in the current study [44,45,46]. Qualitative and quantitative evaluations of bone growth demonstrated no significant differences between the groups. During the initial healing periods (6 and 12 weeks), bone growth in the presence of grafting materials was limited to the periphery of the defect. Nevertheless, at the later stages of the healing process (24 and 48 weeks), a notable expansion of new bone formation was observed, extending from the defect walls towards the center, guided by the xenografts. The grafting materials were shown to act as scaffolds or nucleating sites, thereby promoting bone formation on their respective surfaces [47]. Furthermore, it was observed that both DBB and DPB grafts were integrated into the remodeling bone within the defect site, resulting in complete regeneration of the native anatomic contour after 48 weeks of permitted healing. These findings align with observations in the clinical settings, where both bovine- and porcine-derived xenografts demonstrated sustained bone regeneration [48,49,50,51,52]. Longitudinal studies report that anorganic bovine bone can maintain augmented ridge volume ≥ 5 years, achieving bone stability comparable to autogenous grafts [48,49,50,51]. Similarly, a separate study evaluating implants placed in sites grafted exclusively with porcine xenograft reported high survival rates and minimal marginal bone loss after 5–6 years, underscoring the long-term efficacy of DPB in supporting implant integration [52].

A major structural difference between the DBB and DPB grafts used in this study can be attributed to the presence of a collagen matrix in the former of the two materials [53]. This facilitated improved handling of bovine bone particulates at the intended location, which is consistent with the literature [54]. Contrarily, a deproteinized and sterilized DPB graft was in particulate form without the presence of an aforementioned carrier collagen matrix. Nonetheless, this did not pose a hurdle in the healing process due to the barrier membrane preventing particulate graft dislodgement. While the presence of collagen has been shown to enhance bone formation by improving tissue adhesion and proliferation of osteoblastic cells [55,56], no differences in healing were observed between DBB and DPB grafts. However, additional benefits of the collagen matrix encompassing the DBB grafts relative to particulate DPB are yet to be explored and warrant further investigation.

Volumetric reconstruction analysis of DBB and DPB graft presence (graft (%)) showed no significant differences between 6 and 48 weeks. It has been established that the resorption of deproteinized grafts is a slow process, with some investigations reporting the incorporation of the grafts into the regenerated bone without complete resorption [17,57,58,59,60]. However, the resorption process of xenogeneic biomaterials may be the result of several factors, such as pore size and composition [61,62]. As per the existing literature, DBB and DPB particles presented an overlapping range of macroporosities (ranging from 0.3 mm to 1.5 mm, and between 0.1 mm to 1.0 mm, respectively) [31,63]. Porosity is a key factor in determining the rate of graft resorption and bone ingrowth, as it influences cell migration, nutrient diffusion, and vascular infiltration [64]. Thus, the comparable pore size distributions of DBB and DPB may have facilitated similar patterns of bone remodeling over time. Moreover, anorganic bone from both bovine- and porcine-derived sources are comprised of carbonate apatite and typically possess similar chemical compositions, which could elucidate the reason for similar resorption characteristics among both the experimental groups [31,65,66].

Despite the demonstrated effectiveness of bovine-derived grafts, apprehensions have arisen due to concerns about the transmission of bovine spongiform encephalopathy (BSE). This has prompted a reassessment of its suitability for use [67]. Misfolded proteins, occasionally characterized as infectious particles resulting from BSE in cattle, can exhibit resistance to the sterilization procedures employed in the production of bovine-derived grafts [68,69]. This has also caused several health authorities to develop an internationally well-recognized risk assessment with the production and application of bovine-derived products [70]. On the other hand, DPB grafts are derived from non-transmissible spongiform encephalopathy species that are considered low risk for disease transmission. Furthermore, methodologies to treat DPB particles used have been indicated to eliminate or inactivate viruses and ensure safety for implantation [31,71].

In an effort to further improve healing outcomes, special attention should be paid to the minimization of surgical complications, which is contingent upon the choice of the surgical technique, in addition to biomaterial selection. To mitigate the risk of postoperative complications, it is essential that biomaterials are stabilized and suitably enveloped by the membrane/soft tissue structures, necessitating proficient flap management and suturing. Surgical techniques have also been demonstrated to affect bone morphology during the healing cascade [72,73,74,75]. Of note, minimally invasive techniques, like subperiosteal tunneling, have been shown to be a viable option in the augmentation of deficient lateral alveolar ridges [72,73,74,75]. Additionally, pertaining to surgical instrumentation, debris, carbonization, and thermal damage during osteotomy creation can result in an increase in inflammation, bone resorption, and ultimately cause graft failure [76]. For example, a previous study by Lo Giudice et al. showed that defects induced via ultrasonic surgical instrumentation preserved bone morphology and reduced carbonization relative to more commonly used rotary methods [76]. Therefore, it is essential for surgeons to choose a technique that ensures minimal tissue injury. Likewise, the application of growth factors (in addition to particulate bone grafts) has been recommended in GBR procedures to further hasten osseointegration outcomes [77,78], warranting future preclinical and clinical trials to comparatively evaluate the in vivo performance of grafting materials as a result of changes in surgical instrumentation, surgical techniques, and/or the presence of growth factors.

## 5. Conclusions

The results demonstrate that GBR with DBB or DPB leads to similar trends in bone regeneration from 6 to 48 weeks of permitted healing. More importantly, neither graft material presented an adverse immune response, and healing progressed uneventfully. However, despite the demonstrated effectiveness of bovine-derived grafts, porcine alternatives are characterized under non-transmissible spongiform encephalopathy species and are considered low risk for disease transmission, furthering their suitability for clinical use.

## Figures and Tables

**Table 1:** Summary of the mean  ±  95% CI of bone (%), space (%), and graft (%), within the grafted mandibular defects at 6, 12, 24, and 48 weeks in vivo.

Time (In Vivo)	Group	Bone (%)	Space (%)	Graft (%)
6 weeks	DBB	31.39 ± 8.41	61.91 ± 9.47	6.72 ± 2.17
	DPB	39.76 ± 10.30	51.03 ± 11.60	9.20 ± 2.66
12 weeks	DBB	37.82 ± 8.41	53.71 ± 9.47	8.46 ± 2.17
	DPB	46.11 ± 10.30	44.26 ± 11.60	9.61 ± 2.66
24 weeks	DBB	38.10 ± 8.41	59.29 ± 9.47	2.60 ± 2.17
	DPB	48.54 ± 10.30	46.88 ± 11.60	4.57 ± 2.66
48 weeks	DBB	71.04 ± 8.41	24.24 ± 9.47	4.70 ± 2.17
	DPB	68.38 ± 10.30	23.81 ± 11.60	7.79 ± 2.66

## Data Availability

The raw data supporting the conclusions of this article will be made available by the authors on request.

## References

[1] Chappuis V., Araújo M.G., Buser D. (2017). Clinical relevance of dimensional bone and soft tissue alterations post-extraction in esthetic sites. Periodontology 2000.

[2] Batista M.J., Lawrence H.P., Rosário de Sousa M.d.L. (2014). Impact of tooth loss related to number and position on oral health quality of life among adults. Health Qual. Life outcomes.

[3] Amler M.H., Johnson P.L., Salman I. (1960). Histological and histochemical investigation of human alveolar socket healing in undisturbed extraction wounds. J. Am. Dent. Assoc..

[4] Araújo M.G., Lindhe J. (2005). Dimensional ridge alterations following tooth extraction. An experimental study in the dog. J. Clin. Periodontol..

[5] Atwood D.A., Coy W.A. (1971). Clinical, cephalometric, and densitometric study of reduction of residual ridges. J. Prosthet. Dent..

[6] Hämmerle C.H., Araújo M.G., Simion M. (2012). Evidence-based knowledge on the biology and treatment of extraction sockets. Clin. Oral Implant. Res..

[7] Busei D., Dula K., Belser U.C., Hirt H.-P., Berthold H., Dentistry R. (1995). Localized ridge augmentation using guided bone regeneration. II. Surgical procedure in the mandible. Int. J. Periodontics Restor. Dent..

[8] Buser D., Dula K., Belser U., Hirt H.-P., Berthold H., Dentistry R. (1993). Localized ridge augmentation using guided bone regeneration. I. Surgical procedure in the maxilla. J. Periodontics Restor. Dent..

[9] Zhou X., Zhang Z., Li S., Bai Y., Xu H., Surgery M. (2011). Osteoconduction of different sizes of anorganic bone particles in a model of guided bone regeneration. Br. J. Oral Maxillofac. Surg..

[10] Retzepi M., Donos N. (2010). Guided Bone Regeneration: Biological principle and therapeutic applications. Clin. Oral Implant. Res..

[11] Elgali I., Omar O., Dahlin C., Thomsen P. (2017). Guided bone regeneration: Materials and biological mechanisms revisited. Eur. J. Oral Sci..

[12] Albrektsson T., Johansson C. (2001). Osteoinduction, osteoconduction and osseointegration. Eur. Spine J..

[13] Deligianni D.D., Katsala N.D., Koutsoukos P.G., Missirlis Y.F. (2001). Effect of surface roughness of hydroxyapatite on human bone marrow cell adhesion, proliferation, differentiation and detachment strength. Biomaterials.

[14] Misch C.M. (2010). Autogenous bone: Is it still the gold standard?. Implant Dent..

[15] Schmidt A.H. (2021). Autologous bone graft: Is it still the gold standard?. Injury.

[16] Ferraz M.P. (2023). Bone Grafts in Dental Medicine: An Overview of Autografts, Allografts and Synthetic Materials. Materials.

[17] Gil L.F., Nayak V.V., Benalcázar Jalkh E.B., Tovar N., Chiu K.J., Salas J.C., Marin C., Bowers M., Freitas G., Mbe Fokam D.C.J.J.o.B.M.R.P.B.A.B. (2022). Laddec^®^ versus Bio-Oss^®^: The effect on the healing of critical-sized defect–Calvaria rabbit model. J. Biomed. Mater. Res. Part B: Appl. Biomater..

[18] Shibuya N., Jupiter D.C. (2015). Bone graft substitute: Allograft and xenograft. Clin. Podiatr. Med. Surg..

[19] Rapone B., Inchingolo A.D., Trasarti S., Ferrara E., Qorri E., Mancini A., Montemurro N., Scarano A., Inchingolo A.M., Dipalma G. (2022). Long-Term Outcomes of Implants Placed in Maxillary Sinus Floor Augmentation with Porous Fluorohydroxyapatite (Algipore^®^ FRIOS^®^) in Comparison with Anorganic Bovine Bone (Bio-Oss^®^) and Platelet Rich Plasma (PRP): A Retrospective Study. J. Clin. Med..

[20] Richardson C.R., Mellonig J.T., Brunsvold M.A., McDonnell H.T., Cochran D.L. (1999). Clinical evaluation of Bio-Oss^®^: A bovine-derived xenograft for the treatment of periodontal osseous defects in humans. J. Clin. Periodontol..

[21] Cordaro L., Bosshardt D.D., Palattella P., Rao W., Serino G., Chiapasco M. (2008). Maxillary sinus grafting with Bio-Oss^®^ or Straumann^®^ Bone Ceramic: Histomorphometric results from a randomized controlled multicenter clinical trial. Clin. Oral Implant. Res..

[22] Dai Y., Xu J., Han X.-H., Cui F.-Z., Zhang D.-S., Huang S.-Y. (2021). Clinical efficacy of mineralized collagen (MC) versus anorganic bovine bone (Bio-Oss) for immediate implant placement in esthetic area: A single-center retrospective study. BMC Oral Health.

[23] Piattelli M., Favero G.A., Scarano A., Orsini G., Piattelli A. (1999). Bone reactions to anorganic bovine bone (Bio-Oss) used in sinus augmentation procedures: A histologic long-term report of 20 cases in humans. Int. J. Oral Maxillofac. Implant..

[24] Roldan L., Isaza C., Ospina J., Montoya C., Domínguez J., Orrego S., Correa S. (2023). A Comparative Study of HA/DBM Compounds Derived from Bovine and Porcine for Bone Regeneration. J. Funct. Biomater..

[25] Perić Kačarević Z., Kavehei F., Houshmand A., Franke J., Smeets R., Rimashevskiy D., Wenisch S., Schnettler R., Jung O., Barbeck M. (2018). Purification processes of xenogeneic bone substitutes and their impact on tissue reactions and regeneration. Int. J. Artif. Organs.

[26] Pereira R.d.S., de Carvalho M.V.N.B., Hochuli-Vieira E., Statkievicz C., Pereira Santos D.L., Augusto Neto R.T., Pinto C.d.F.S., Bennardo F., Mourão C.F.J.M. (2024). Histomorphometric and Micro-CT Evaluation of Cerabone and Bio-Oss in Maxillary Sinus Lifting: A Randomized Clinical Trial. Medicina.

[27] Hu L., Xiang S., Liu J., Ye L., Cao Z., Pan J.J.O.S. (2022). Combigraft versus Bio-Oss/Bio-Gide in alveolar ridge preservation: A prospective randomized controlled trial. Oral Surg..

[28] Titsinides S., Agrogiannis G., Karatzas T. (2019). Bone grafting materials in dentoalveolar reconstruction: A comprehensive review. Jpn. Dent. Sci. Rev..

[29] Seo Y.H., Hwang S.H., Kim Y.N., Kim H.J., Bae E.B., Huh J.B. (2022). Bone Reconstruction Using Two-Layer Porcine-Derived Bone Scaffold Composed of Cortical and Cancellous Bones in a Rabbit Calvarial Defect Model. Int. J. Mol. Sci..

[30] Singh R., Mahesh L., Shukla S.J.I.J.O.I.C.R. (2013). Infections resulting from bone grafting biomaterials. Int. J. Oral Implant. Clin. Res..

[31] Collagen Matrix I. Dental Repair Solutions. 15. https://regenity.com/solution/dental/.

[32] Bergamo E.T.P., Balderrama Í.d.F., Ferreira M.R., Spielman R., Slavin B.V., Torroni A., Tovar N., Nayak V.V., Slavin B.R., Coelho P.G. (2023). Osteogenic differentiation and reconstruction of mandible defects using a novel resorbable membrane: An in vitro and in vivo experimental study. J. Biomed. Mater. Res. Part B Appl. Biomater..

[33] Benic G.I., Hammerle C.H. (2014). Horizontal bone augmentation by means of guided bone regeneration. Periodontology 2000.

[34] Dimitriou R., Mataliotakis G.I., Calori G.M., Giannoudis P.V. (2012). The role of barrier membranes for guided bone regeneration and restoration of large bone defects: Current experimental and clinical evidence. BMC Med..

[35] Hammerle C.H., Jung R.E. (2003). Bone augmentation by means of barrier membranes. Periodontology 2000.

[36] Khan R.S., Aslam M., Ucer C., Wright S. (2023). Success of Xenografts in Alveolar Ridge Preservation Based on Histomorphometric Outcomes. Dent. J..

[37] Kantarci A., Hasturk H., Van Dyke T.E. (2015). Animal models for periodontal regeneration and peri-implant responses. Periodontology 2000.

[38] Sennerby L., Persson L.G., Berglundh T., Wennerberg A., Lindhe J.J.C.I.D., Research R. (2005). Implant stability during initiation and resolution of experimental periimplantitis: An experimental study in the dog. Clin. Implant. Dent. Relat. Res..

[39] Gotfredsen K., Berglundh T., Lindhe J. (2002). Bone reactions at implants subjected to experimental peri-implantitis and static load: A study in the dog. J. Clin. Periodontol..

[40] Pellegrini G., Seol Y.J., Gruber R., Giannobile W.V. (2009). Pre-clinical models for oral and periodontal reconstructive therapies. J. Dent. Res..

[41] Miyauchi Y., Izutani T., Teranishi Y., Iida T., Nakajima Y., Xavier S.P., Baba S. (2022). Healing Patterns of Non-Collagenated Bovine and Collagenated Porcine Xenografts Used for Sinus Floor Elevation: A Histological Study in Rabbits. J. Funct. Biomater..

[42] Hwang S.H., Moon K., Du W., Cho W.T., Huh J.B., Bae E.B. (2023). Effect of Porcine- and Bovine-Derived Xenografts with Hydroxypropyl Methylcellulose for Bone Formation in Rabbit Calvaria Defects. Materials.

[43] Lai V.J., Michalek J.E., Liu Q., Mealey B.L. (2020). Ridge preservation following tooth extraction using bovine xenograft compared with porcine xenograft: A randomized controlled clinical trial. J. Periodontol..

[44] Schwarz F., Herten M., Ferrari D., Wieland M., Schmitz L., Engelhardt E., Becker J. (2007). Guided bone regeneration at dehiscence-type defects using biphasic hydroxyapatite+beta tricalcium phosphate (Bone Ceramic^®^) or a collagen-coated natural bone mineral (BioOss Collagen^®^): An immunohistochemical study in dogs. Int. J. Oral Maxillofac. Surg..

[45] Park J.-I., Yang C., Kim Y.-T., Kim M.-S., Lee J.-S., Choi S.-H., Jung U.-W. (2014). Space maintenance using crosslinked collagenated porcine bone grafted without a barrier membrane in one-wall intrabony defects. J. Biomed. Mater. Res. Part B Appl. Biomater..

[46] Park S.J., Rahman M.M., Lee J., Kang S.W., Kim S. (2023). Investigation of Bone Regeneration Efficacy of New Bovine Bone Minerals in a Canine Mandibular Critical Defect Model. Adv. Healthc. Mater..

[47] Bee S.L., Hamid Z.A.A.J.J.o.B.M.R.P.B.A.B. (2022). Asymmetric resorbable-based dental barrier membrane for periodontal guided tissue regeneration and guided bone regeneration: A review. J. Biomed. Mater. Res. Part B Appl. Biomater..

[48] Juodzbalys G., Raustia A.M., Kubilius R. (2007). A 5-year follow-up study on one-stage implants inserted concomitantly with localized alveolar ridge augmentation. J. Oral Rehabil..

[49] Beretta M., Cicciù M., Poli P.P., Rancitelli D., Bassi G., Grossi G.B., Maiorana C. (2015). A Retrospective Evaluation of 192 Implants Placed in Augmented Bone: Long-Term Follow-Up Study. J. Oral Implantol..

[50] Schmitt C.M., Moest T., Lutz R., Neukam F.W., Schlegel K.A. (2015). Anorganic bovine bone (ABB) vs. autologous bone (AB) plus ABB in maxillary sinus grafting. A prospective non-randomized clinical and histomorphometrical trial. Clin. Oral Implant. Res..

[51] Lutz R., Berger-Fink S., Stockmann P., Neukam F.W., Schlegel K.A. (2015). Sinus floor augmentation with autogenous bone vs. a bovine-derived xenograft—A 5-year retrospective study. Clin. Oral Implant. Res..

[52] Choi J.W., Hwang S.S., Yun P.Y., Kim Y.K. (2023). Horizontal ridge augmentation with porcine bone-derived grafting material: A long-term retrospective clinical study with more than 5 years of follow-up. J. Korean Assoc. Oral Maxillofac. Surg..

[53] Araújo M.G., Lindhe J. (2009). Ridge preservation with the use of Bio-Oss^®^ collagen: A 6-month study in the dog. Clin. Oral Implant. Res..

[54] Mario Roccuzzo D.J.I.J.P.R.D. (2014). Long-term stability of soft tissues following alveolar ridge preservation: 10-year results of a prospective study around nonsubmerged implants. Int. J. Periodontics Restor. Dent..

[55] Ignatius A., Blessing H., Liedert A., Schmidt C., Neidlinger-Wilke C., Kaspar D., Friemert B., Claes L. (2005). Tissue engineering of bone: Effects of mechanical strain on osteoblastic cells in type I collagen matrices. Biomaterials.

[56] Gresita A., Raja I., Petcu E., Hadjiargyrou M. (2023). Collagen-Coated Hyperelastic Bone Promotes Osteoblast Adhesion and Proliferation. Materials.

[57] Paknejad M., Rokn A.R., Yaghobee S., Moradinejad P., Heidari M., Mehrfard A.J.J.o.D. (2014). Effects of two types of anorganic bovine bone on bone regeneration: A histological and histomorphometric study of rabbit calvaria. J. Dent..

[58] Schliephake H., Dard M., Planck H., Hierlemann H., Jakob A. (2000). Guided bone regeneration around endosseous implants using a resorbable membrane vs a PTFE membrane. Clin. Oral Implant. Res..

[59] Hämmerle C.H., Brägger U., Bürgin W., Lang N.P. (1996). The effect of subcrestal placement of the polished surface of ITI implants on marginal soft and hard tissues. Clin. Oral Implant. Res..

[60] Avera S.P., Stampley W.A., McAllister B.S. (1997). Histologic and clinical observations of resorbable and nonresorbable barrier membranes used in maxillary sinus graft containment. Int. J. Oral Maxillofac. Implant..

[61] Lee J.H., Yi G.S., Lee J.W., Kim D.J. (2017). Physicochemical characterization of porcine bone-derived grafting material and comparison with bovine xenografts for dental applications. J. Periodontal Implant. Sci..

[62] Pimentel I., Henriques B., Silva F., Carvalho O., Teughels W., Ozcan M., Souza J.C.M. (2023). Morphological aspects and distribution of granules composed of deproteinized bovine bone or human dentin into a putty mixture: An in vitro study. Head Face Med..

[63] Tapety F.I., Amizuka N., Uoshima K., Nomura S., Maeda T. (2004). A histological evaluation of the involvement of Bio-Oss^®^ in osteoblastic differentiation and matrix synthesis. Clin. Oral Implant. Res..

[64] Mukasheva F., Adilova L., Dyussenbinov A., Yernaimanova B., Abilev M., Akilbekova D. (2024). Optimizing scaffold pore size for tissue engineering: Insights across various tissue types. Front. Bioeng. Biotechnol..

[65] Figueiredo M., Fernando A., Martins G., Freitas J., Judas F., Figueiredo H. (2010). Effect of the calcination temperature on the composition and microstructure of hydroxyapatite derived from human and animal bone. Ceram. Int..

[66] Mano T., Akita K., Fukuda N., Kamada K., Kurio N., Ishikawa K., Miyamoto Y. (2020). Histological comparison of three apatitic bone substitutes with different carbonate contents in alveolar bone defects in a beagle mandible with simultaneous implant installation. J. Biomed. Mater. Res. Part B Appl. Biomater..

[67] Wenz B., Oesch B., Horst M. (2001). Analysis of the risk of transmitting bovine spongiform encephalopathy through bone grafts derived from bovine bone. Biomaterials.

[68] Moreno-Gonzalez I., Soto C. (2011). Misfolded protein aggregates: Mechanisms, structures and potential for disease transmission. Semin. Cell Dev. Biol..

[69] Scicchitano L.J.I. (2004). Bovine spongiform encephalopathy and Creutzfeldt-Jakob disease: Background and implications for nursing practice. Insight.

[70] Sogal A., Tofe A.J.J.o.p. (1999). Risk assessment of bovine spongiform encephalopathy transmission through bone graft material derived from bovine bone used for dental applications. J. Periodontol..

[71] Li S.-T., Chen H.-C., Yuen D. (2015). Method of Preparing Porous Carbonate Apatite from Natural Bone. https://patents.google.com/patent/US20150250921A1/en.

[72] De Stavola L., Tunkel J., Dentistry R. (2013). Results of vertical bone augmentation with autogenous bone block grafts and the tunnel technique: A clinical prospective study of 10 consecutively treated patients. Int. J. Periodontics Restor. Dent..

[73] Sanz-Sánchez I., Sanz-Martín I., Ortiz-Vigón A., Molina A., Sanz M. (2022). Complications in bone-grafting procedures: Classification and management. Periodontology 2000.

[74] Hasson O. (2007). Augmentation of deficient lateral alveolar ridge using the subperiosteal tunneling dissection approach. Oral Surg. Oral Med. Oral Pathol. Oral Radiol. Endodontol..

[75] Nevins M.L., Camelo M., Nevins M., Schupbach P., Friedland B., Camelo J.M.B., Kim D.M.J.I.J.o.P., Dentistry R. (2009). Minimally invasive alveolar ridge augmentation procedure (tunneling technique) using rhPDGF-BB in combination with three matrices: A case series. Int. J. Periodontics Restor. Dent..

[76] Lo Giudice R., Puleio F., Rizzo D., Alibrandi A., Lo Giudice G., Centofanti A., Fiorillo L., Di Mauro D., Nicita F. (2019). Comparative Investigation of Cutting Devices on Bone Blocks: An SEM Morphological Analysis. Appl. Sci..

[77] Zhu L., Du X., Fu G., Wang L., Huang H., Wu X., Xu B.J.B.O.H. (2025). Efficacy of different forms of concentrated growth factors combined with deproteinized bovine bone minerals in guided bone regeneration: A randomized clinical trial. BMC Oral Health.

[78] Fiorillo L., Cervino G., Galindo-Moreno P., Herford A.S., Spagnuolo G., Cicciù M.J.B.R.I. (2021). Growth factors in oral tissue engineering: New perspectives and current therapeutic options. BioMed Res. Int..

